# Deletion in chromosome 6 spanning alpha-synuclein and multimerin1 loci in the Rab27a/b double knockout mouse

**DOI:** 10.1038/s41598-022-13557-8

**Published:** 2022-06-14

**Authors:** Rudradip Pattanayak, Rachel Underwood, Michael R. Crowley, David K. Crossman, Jennifer R. Morgan, Talene A. Yacoubian

**Affiliations:** 1 Center for Neurodegeneration and Experimental Therapeutics, Department of Neurology, Heersink School of Medicine, University of Alabama at Birmingham, 1719 Sixth Avenue South, Civitan International Research Building 510A, Birmingham, AL 35294 USA; 2 Department of Genetics, Heersink School of Medicine, University of Alabama at Birmingham, Birmingham, AL 35294 USA; 3 The Eugene Bell Center for Regenerative Biology and Tissue Engineering, Marine Biological Laboratory, Woods Hole, MA 02543 USA

**Keywords:** Biological techniques, Animal disease models, Genetic models, Cell biology, Neuroscience, Diseases of the nervous system

## Abstract

We report an incidental 358.5 kb deletion spanning the region encoding for alpha-synuclein (αsyn) and multimerin1 (Mmrn1) in the Rab27a/Rab27b double knockout (DKO) mouse line previously developed by Tolmachova and colleagues in 2007. Western blot and RT-PCR studies revealed lack of αsyn expression at either the mRNA or protein level in Rab27a/b DKO mice. PCR of genomic DNA from Rab27a/b DKO mice demonstrated at least partial deletion of the *Snca* locus using primers targeted to exon 4 and exon 6. Most genes located in proximity to the *Snca* locus, including *Atoh1*, *Atoh2*, *Gm5570*, *Gm4410*, *Gm43894*, and *Grid2*, were shown not to be deleted by PCR except for *Mmrn1*. Using whole genomic sequencing, the complete deletion was mapped to chromosome 6 (60,678,870–61,037,354), a slightly smaller deletion region than that previously reported in the C57BL/6J substrain maintained by Envigo. Electron microscopy of cortex from these mice demonstrates abnormally enlarged synaptic terminals with reduced synaptic vesicle density, suggesting potential interplay between Rab27 isoforms and αsyn, which are all highly expressed at the synaptic terminal. Given this deletion involving several genes, the Rab27a/b DKO mouse line should be used with caution or with appropriate back-crossing to other C57BL/6J mouse substrain lines without this deletion.

## Introduction

Rab GTPases are a conserved family of proteins with over 60 mammalian members that are important to protein trafficking in cells^1–4^. These Rab proteins function through a catalytic GTP/GDP binding site, which, when GTP bound, leads to a conformational switch promoting interaction with its effector proteins^2,4–7^. Rab27a and Rab27b are two highly homologous Rab GTPases that regulate vesicle trafficking in a wide variety of cell types. Rab27a and Rab27b have been linked to exocytosis in specialized secretory cells, including melanocytes, cytotoxic T cells, platelets, neutrophils, mast cells, and pancreatic acinar cells, among others^5,8–19^. In the brain, these Rab GTPases have been linked to axonal trafficking and synaptic function^20–23^. Rab27s have also been implicated in endocytic processes in pancreatic beta-islet cells^24–26^.

Much of the functional roles for Rab27a and Rab27b have been identified through studies using a Rab27a/b double knockout (DKO) mouse line created by Tolmachova et al*.*^13^. This group first created a Rab27b null line (Rab27b^−/−^) using Cre-loxP site recombination, and then subsequently crossed this Rab27b null line with a naturally occurring Rab27a knockout (KO) mice line (designated as *ashen,* Rab27a^ash/ash^), thereby generating Rab27a^ash/ash^/Rab27b^−/−^ mice (Rab27 DKO)^13^. This Rab27 DKO line was generated and maintained in the C57BL/6J mouse strain^13^. The Rab27 DKO mouse line was originally used to demonstrate Rab27s’ effects on the number and secretion of platelet dense granules^13^. This Rab27 DKO mouse has also been used to show Rab27s’ role in lung epithelial cell secretion^27^, neutrophil primary granule exocytosis and chemotaxis^10^, exosomal secretion^28^, mast cell granule secretion^11^, and lacrimal gland cell secretion^29^, among other functions.

We recently became interested in the potential role of Rab27s in Parkinson’s disease (PD) and Dementia with Lewy Bodies (DLB), two neurodegenerative disorders that are marked by pathological cytoplasmic aggregates, termed Lewy Bodies, which are highly enriched in alpha-synuclein (αsyn)^30^. GWAS studies have shown that certain SNPs in αsyn are associated with increased risk of PD and DLB^31–37^, and certain mutations in αsyn are genetic causes of familial forms of PD^38–41^. αSyn is highly expressed near the synaptic vesicles in the presynaptic axon terminals and likely plays a role in modulating synaptic neurotransmission^42–44^. Rab27a and Rab27b regulate synaptic vesicle (SV) exocytosis and recycling at synaptic terminals, where they could interact with αsyn^21–23^. Rab27b is particularly highly expressed in neurons in key brain areas affected in PD and DLB^8^. Our group recently discovered elevated Rab27b levels in an αsyn overexpression in vitro model and in postmortem brain tissue from PD and DLB patients^45^. We also demonstrated that shRNA mediated knockdown of Rab27b increased αsyn insolubility and toxicity by disrupting autophagic flux in an inducible αsyn paracrine model^45^.

In order to examine the role of Rab27a/b in αsyn trafficking and aggregation, we obtained the Rab27 DKO mouse line generated by Tolmachova et al.^13^. In the characterization of the Rab27 DKO mouse line, we found a surprising absence of αsyn mRNA and protein expression in the brain tissue of Rab27 DKO mice. To understand the mechanism for lack of αsyn expression in the brain, we performed genomic analysis of these mice and found the presence of a spontaneous ~ 360 kb deletion involving the loci for *Snca* and *Mmrn1*, which encode for αsyn and multimerin 1, respectively, in the Rab27 DKO mouse line. Ultrastructural analysis of these mice demonstrated abnormal synaptic terminals with increased bouton area and reduced synaptic vesicle number and density, suggesting a possible interplay between Rab27s and αsyn in the regulation of synaptic function.

## Results

### Rab27 DKO mice lack αsyn mRNA and protein expression in the cortex and hippocampus

**Figure 1:**
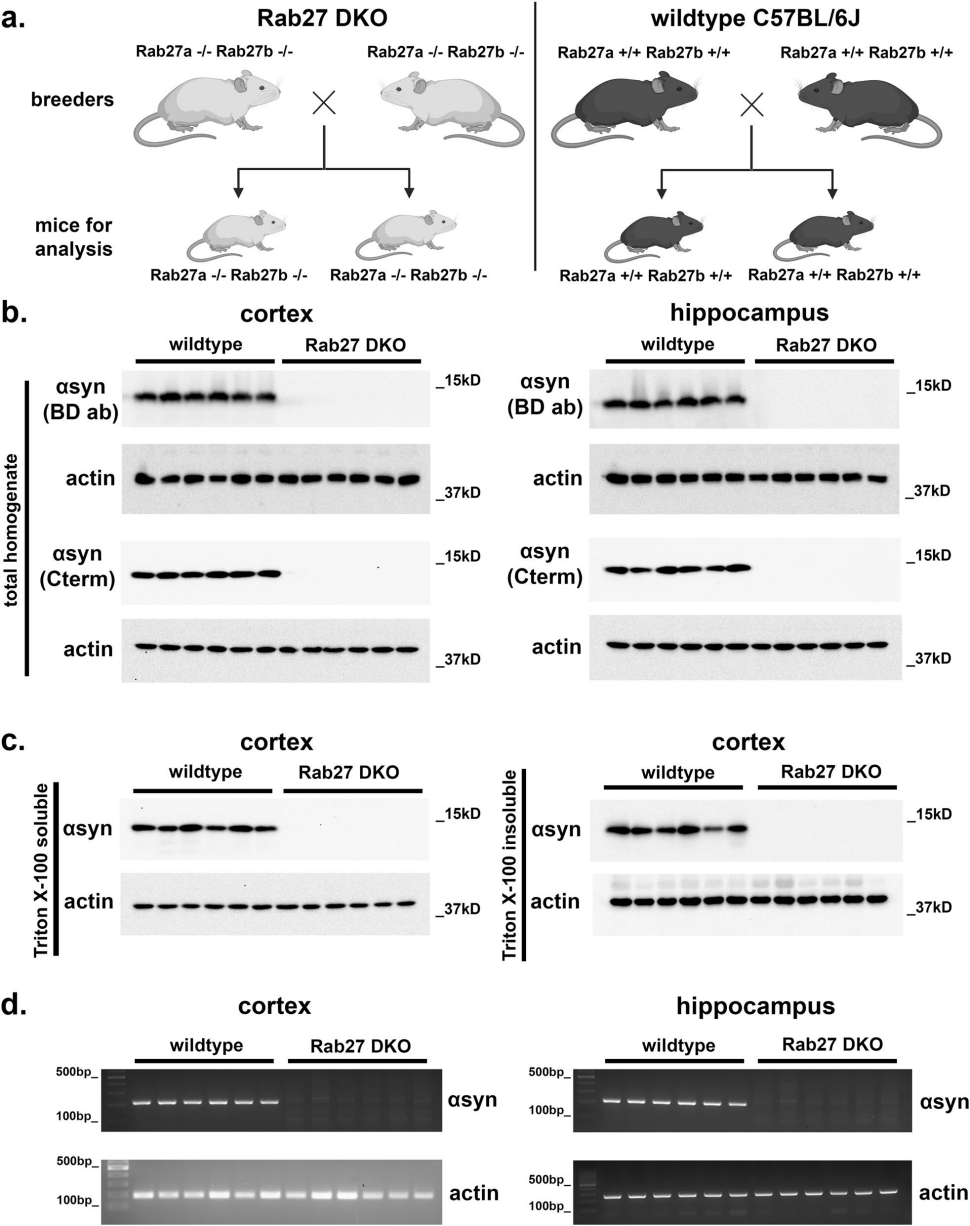
Rab27 DKO mouse brains lack αsyn expression. (**a**) Breeding scheme for Rab27 DKO mice and wildtype C57BL/6J mice used for this study. Briefly, Rab27 DKO mice were bred in homozygous non-litter mate pairs. Since the Rab27 DKO mice were generated in the C57BL/6J strain, we compared the Rab27 DKO mice to C57BL/6J mice obtained from Jackson Laboratories. (**b**) Total homogenates from the cortex and hippocampus of 4 week-old wildtype C57BL/6J mice and Rab27 DKO mice were analyzed by Western blot using two different αsyn antibodies, one directed at an epitope within the protein (BD αsyn antibody #610787) and another one directed at a C-terminal epitope (Cell Signaling αsyn antibody #2642S). N = 6 per genotype. (**c**) Triton X-100 soluble and Triton X-100 insoluble lysates from the cortex and hippocampus of 4 week-old wildtype C57BL/6J mice and Rab27 DKO mice were analyzed by Western blot. N = 6 per genotype. (**d**) RNA samples from the cortex and hippocampus of 4 week-old wildtype C57BL/6J mice and Rab27 DKO mice were examined for αsyn mRNA expression by RT-PCR. N = 6 per genotype.

The initial Rab27 DKO mice we obtained were bred to each other for several generations initially, prior to plans to breed to C57BL6/J mice in order to separate out the Rab27a KO from the Rab27b KO and to generate rederived “control” C57BL/6J animals. To characterize αsyn expression in these Rab27 DKO mice, we measured αsyn protein expression in mouse brain lysates from 4 week old Rab27 DKO mice by Western blot analysis and compared these brain lysates to that from wildtype C57BL/6J mice obtained from Jackson Labs, as the Rab27 DKO mouse line had been generated and maintained in C57BL/6J strain (Fig. 1a). While a strong signal of monomeric αsyn was detected in both cortical or hippocampal lysates from male and female wildtype mice, no αsyn was detectable in total homogenates from the cortex or hippocampus from male or female Rab27 DKO mice (Fig. 1b; Supp. Fig. 1). We next performed Western blot analysis on the cortical samples from wildtype and Rab27 DKO mice using a different anti-αsyn antibody directed against the C-terminal epitope (Fig. 1b; Supp. Fig. 1). Similarly, no αsyn was detected in the Rab27 DKO mice, suggesting that the lack of αsyn detection was unlikely due to lack of detection of a hidden epitope by the first antibody. To test whether αsyn was found in the insoluble fractions instead, we then measured αsyn protein levels in Triton X-100 soluble and Triton X-100 insoluble fractions and similarly observed an absence of αsyn in either fraction in the hippocampus or cortex of Rab27 DKO mice compared to wildtype mice (Fig. 1c; Supp. Fig. 1).

To rule out the possibility of Rab27 DKO-induced abnormalities in αsyn protein translation or a shift in αsyn aggregation state that may limit detection by the anti-αsyn antibodies used for Western blot, we next measured αsyn mRNA by RT-PCR. While αsyn mRNA was easily detectable by RT-PCR in the cortex and hippocampus from wildtype C57BL/6J mice, we were unable to detect αsyn mRNA expression in the cortex or hippocampus of Rab27 DKO mice (Fig. 1d; Supp. Fig. 2).

### Rab27 DKO mice demonstrate genomic deletion involving the *Snca* locus

**Figure 2:**
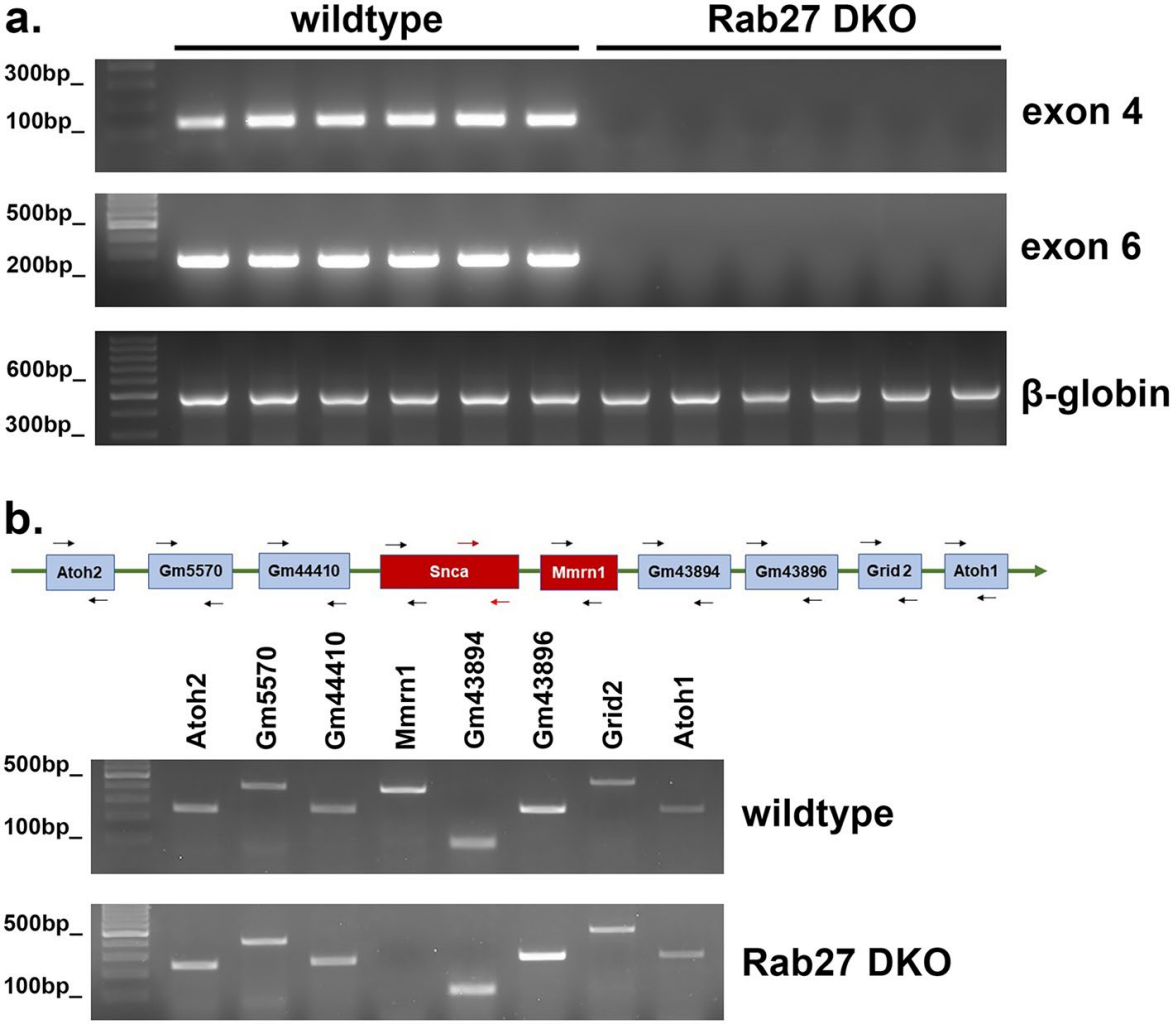
Rab27 DKO mice lack *Snca* and *Mmrn1* loci on chromosome 6. (**a**) PCR with primers directed against exon 4 and exon 6 of *Snca* was performed using genomic DNA from wildtype C57BL/6J mice and Rab27 DKO mice. N = 6 per genotype. (**b**) PCR with primers directed at multiple loci surrounding the *Snca* locus on chromosome 6 was performed using genomic DNA from wildtype C57BL/6J mice and Rab27 DKO mice.

The lack of any detectable αsyn by Western blot or RT-PCR suggested a previously uncharacterized deletion of the *Snca* locus in this Rab27 DKO mouse line. We prepared genomic DNA from wildtype C57BL/6J mice and Rab27 DKO mice and then performed PCR using two different sets of *Snca* primers, one directed to *Snca* exon 4 and one directed to *Snca* exon 6^46^. Neither *Snca* exon 4 nor exon 6 were present in genomic DNA from Rab27 DKO mice, whereas wildtype mice demonstrated presence of the *Snca* locus (Fig. 2a; Supp. Fig. 3). PCR against beta-globin confirmed the presence of intact genomic DNA from Rab27 DKO mice (Fig. 2a; Supp. Fig. 3). Based on this PCR, we concluded that at least part of the *Snca* locus was deleted in Rab27 DKO mice.

To examine the extent of the deletion region, we also performed PCR for several genes surrounding the *Snca* locus on chromosome 6, including *Atoh2*, *Gm5570*, *Gm44410*, *Mmrn1*, *Gm43894*, *Gm43896*, *Grid2*, *Gm44072*, and *Atoh1*. All surrounding loci were detectable by PCR using genomic DNA from wildtype mice (Fig. 2b; Supp. Fig. 3). Genomic DNA from Rab27 DKO mice also demonstrated the presence of the loci for *Atoh2*, *Gm5570*, *Gm44410*, *Gm43894*, *Gm43896*, *Grid2*, *Gm44072*, and *Atoh1*. However, Rab27 DKO mice lacked the *Mmrn1* loci, the gene most proximal to the *Snca* locus (Fig. 2b; Supp. Fig. 3).

**Figure 3:**
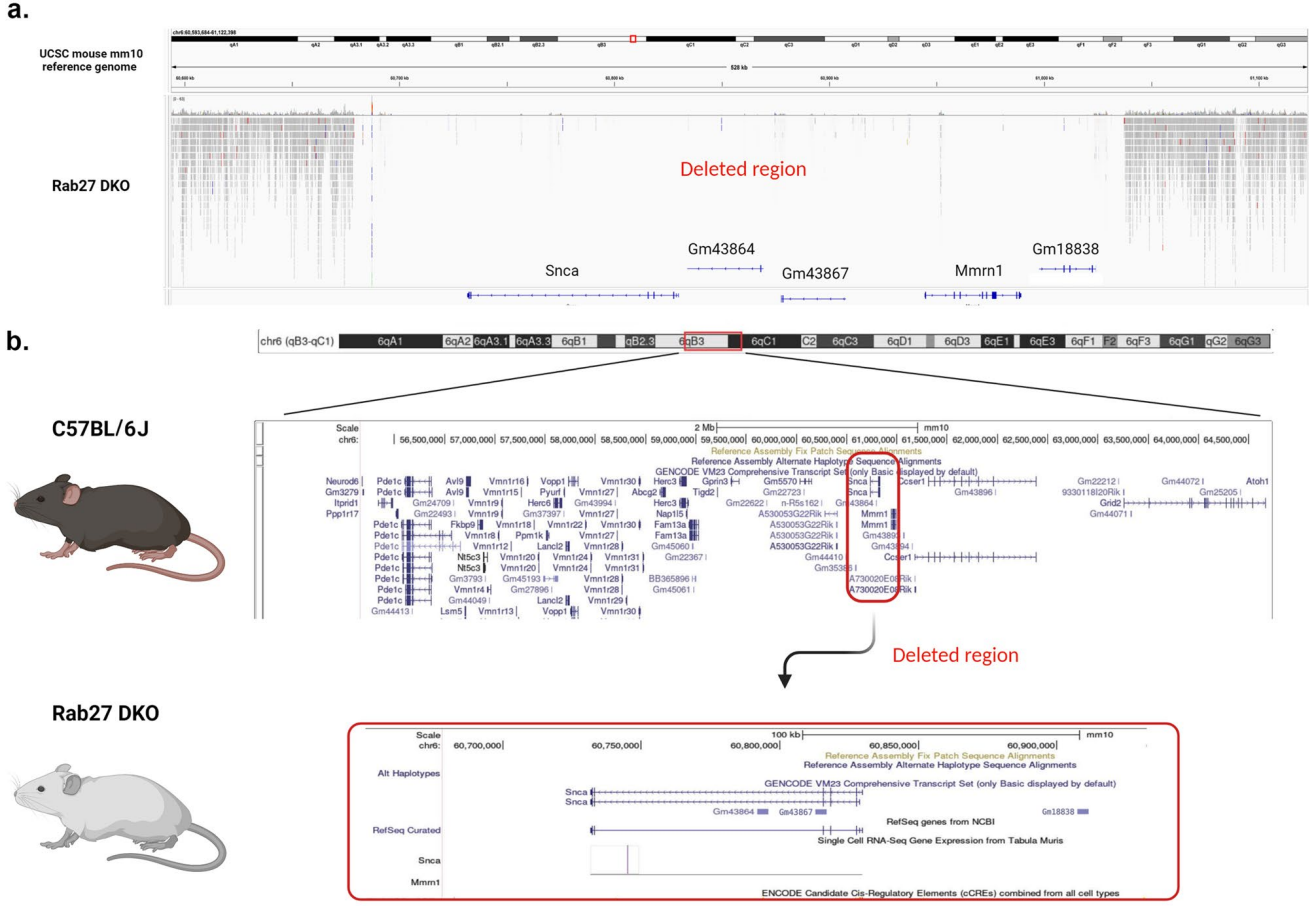
Rab27 DKO mice have a ~ 360 kb deletion on chromosome 6. (**a**) Representative view for the deletion region from the Integrative Genomics Viewer (IGV) for the Rab27 DKO mice aligned to the UCSC mouse reference genome mm10. (**b**) Model demonstrates deletion region involving the *Snca* and *Mmrn1* loci on chromosome 6 in the Rab27 DKO mice.

We next performed whole genome sequencing to determine the extent of the deletion region in these Rab27 DKO mice. Upon comparison of the Rab27 DKO genomic sequencing data with the genome sequence of UCSC mouse GRCm38/mm10 reference genome, the deletion on chromosome 6 was found to extend from 60,678,870 to 61,037,354 in Rab27 DKO mice. This region contains both the *Snca* and *Mmrn1* loci (Fig. 3; supplementary sequencing files). Additional predicted genes in this region that were deleted in the Rab27 DKO mouse included Gm43864, Gm43867, and Gm18838.

### Rab27 DKO mice with additional *Snca* and *Mmrn1* genomic deletion demonstrate abnormal synaptic architecture

**Figure 4:**
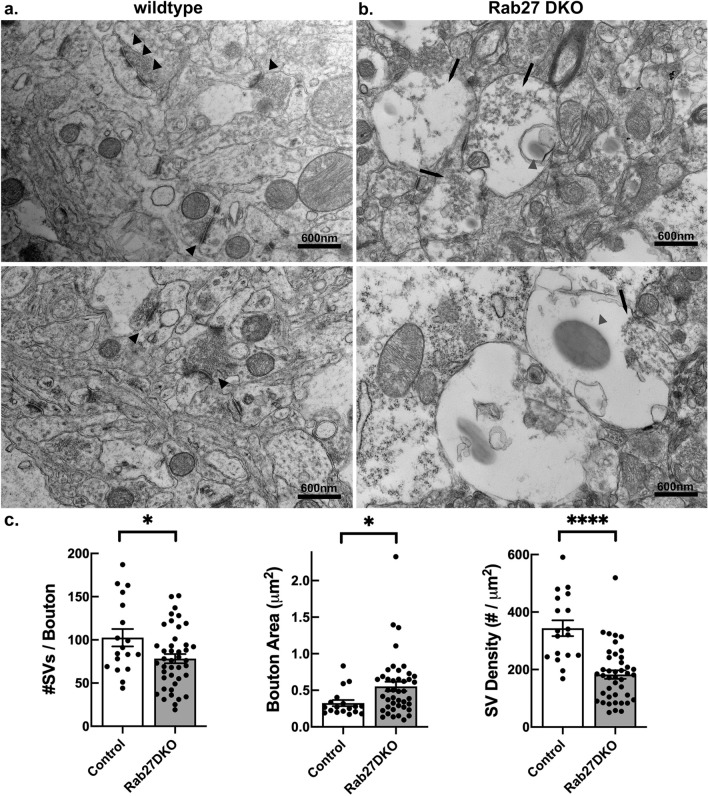
Rab27 DKO mice demonstrate abnormal synaptic terminal ultrastructure. (**a**) Representative transmission EM images of motor cortex from a wildtype C57BL/6J mouse. Scale bar = 600 nm. Black arrowheads point to synaptic terminals. (**b**) Representative transmission EM images of motor cortex from a Rab27 DKO mouse with the chromosome 6 deletion involving *Snca* and *Mmrn1*. Scale bar = 600 nm. Black arrows point to abnormal synaptic terminals. Gray arrowheads point to membrane-less structures not observed in wildtype mice. (**c**) Quantification of synaptic vesicle number, bouton area, and synaptic vesicle density in motor cortices from wildtype C57BL/6J and Rab27 DKO mice with the chromosome 6 deletion involving *Snca* and *Mmrn1*. n = 18 synapses for control and 42 synapses for Rab27 DKO. *p < 0.05, ****p < 0.0001 (unpaired, two-tailed *t* test).

Since Rab27a, Rab27b, and αsyn are enriched in synaptic terminals in the brain and have been implicated in synaptic vesicle trafficking, we performed transmission electron microscopy of motor cortex from wildtype C57BL/6J mice and Rab27 DKO mice. While wildtype mouse cortex showed normal ultrastructure (Fig. 4a), Rab27 DKO mouse cortex demonstrated frequent swollen synaptic boutons with abnormal synaptic vesicle organization (Fig. 4b). Synapses from Rab27 DKO mice showed reduced synaptic vesicle numbers (Fig. 4c; unpaired, two-tailed *t* test t_58_ = 2.310; p = 0.0245) and increased bouton area (Fig. 4c; unpaired, two-tailed *t* test t_58_ = 2.243; p = 0.0287). As a result, synaptic vesicle density in Rab27 DKO mice was reduced to nearly half that in wildtype C57BL/6J mice (Fig. 4c; unpaired, two-tailed *t* test t_58_ = 5.59; p < 0.0001). Additionally, these Rab DKO brains revealed strange membrane-less oval structures surrounded by empty space suggestive of lipid droplets (Fig. 4b).

## Discussion

Due to the potential role of Rab27s in synucleinopathies such as PD and DLB, we examined the expression of αsyn in the Rab27 DKO mouse developed by Tolmachova et al.^13^. Surprisingly, we observed lack of both protein and mRNA expression for αsyn in both the cortex and hippocampus from Rab27 DKO mice. Further investigation showed that both the *Snca* locus and the nearby *Mmrn1* locus were not detected in genomic DNA from Rab27 DKO mice. Genome wide sequencing confirmed a deletion of 358.5 kb on mouse chromosome 6 extending from 60,678,870 to 61,037,354, a region that encompasses both the *Snca* and *Mmrn1* loci along with Gm43864, Gm43867, and Gm18838. These mice demonstrated abnormal synaptic structure in the motor cortex.

A very similar genomic deletion on chromosome 6 extending from 60.976 to 61.341 Mb has been previously reported in a substrain of C57BL/6J mouse line maintained in the UK (C57BL/6JOlaHsd)^47^. This C57BL/6J substrain was originally obtained from the Jackson Laboratory in 1983 and has been maintained at Harlan Laboratories, now Envigo Laboratories^46,48,49^. The chromosomal deletion was thought present in the Harlan Laboratory as early as 1997^46,48,49^. Although this deletion is not identical to the one we have described here in Rab27 DKO mice, it is highly similar. We suspect that, as the Rab27 DKO mouse line was originally created in the UK, it is very likely that it may have been produced in the C57BL/6JOlaHsd substrain. Slight differences in the genomic deletion may be a result of genetic drift and instability over time.

This Rab27 DKO mouse line has been used to implicate Rab27a and Rab27b in several exocytic functions, including platelet granule secretion, neutrophil granule exocytosis, lung epithelial secretion, exosome secretion, and lacrimal gland secretion^10,11,13,27–29^. Depending on the breeding methods used to maintain the Rab27 DKO line in different mouse colonies, deletion of the region involving both *Snca* and *Mmrn1* loci on chromosome 6 is not necessarily present in all Rab27 DKO mice. However, a re-evaluation and re-interpretation of data generated using this Rab27 DKO line is indicated. αSyn has primarily been associated with neurodegenerative disorders, such as PD and DLB, and is highly expressed in neurons. However, other cells that express αsyn include red blood cells, platelets, T cells, B cells, NK cells, and monocytes^50^. It is possible that abnormalities in platelet function^13^ and/or exosome secretion^28^ observed in the Rab27 DKO mouse could also be related to αsyn deletion in this mouse. Indeed, αsyn KO mice demonstrate abnormal platelet function^50^.

Multimerin 1, a member of the EMILIN protein family of disulfide-linked multimeric proteins, is a large, soluble protein with limited expression in platelets and endothelial cells^51^. Multimerin 1 promotes adhesion of platelets, endothelial cells, neutrophils, smooth muscle cells, and fibroblasts, and its primary function is regulation of coagulation^51,52^. *Mmrn1* deletion could also contribute to the phenotypic findings of platelet and neutrophil abnormalities observed in the Rab27 DKO mouse^10,13^. Indeed, impaired platelet adhesion and thrombus formation observed in the C57BL/6JOlaHsd substrain was shown to be rescued by recombinant multimerin 1 infusion^53^. Interestingly, multimerin 1 expression is increased in αsyn KO mouse^54^. Other predicted genes in this region, such as Gm43864, Gm43867, and Gm18838, could also contribute to the findings attributed to the Rab27 DKO phenotype.

Our examination of the Rab27 DKO mouse brain points to a potential overlap of Rab27a, Rab27b, and αsyn function in the brain. Rab27a, Rab27b, and αsyn are enriched in the synaptic terminals in neurons, and these proteins have been implicated in synaptic vesicle trafficking^21–23,42–44^. Using TEM of motor cortex from Rab27 DKO mice with the additional deletion involving the *Snca* locus, we observed abnormal synaptic boutons with a reduction in synaptic vesicle number and density and an increase in bouton area. These abnormalities are possibly more pronounced that those described previously in either αsyn KO mice^42,44^ or Rab27 KO mouse developed by Gomi et al*.*^8^. Pavlos et al*.* previously observed that Rab27b is highly expressed at the synaptic terminal and inhibition of Rab27b diminishes synaptic vesicle recycling in mouse hippocampal neurons^22^. Similarly, subtle changes in synaptic vesicle recycling and transmission have been described in invertebrate Rab27 KO models (Yu ref)^21^. Other groups have shown a key function for αsyn at the synapse, with knockdown or knockout of αsyn affecting the generation or maintenance of the reserve synaptic vesicle pool^42–44^. Triple KO of αsyn, βsyn, and γsyn causes reduction in presynaptic terminal size, and alterations in synaptic vesicle pool organization and synaptic transmission^55,56^.

Given the individual effects of Rab27 KO and αsyn KO on synaptic organization, loss of Rab27s and αsyn together could thus additively or synergistically contribute to the abnormal synapse structures we observed in the Rab27 DKO mice with the *Snca/Mmrn1* deletion. Deletion of *Mmrn1* is unlikely to contribute to this finding, as Multimerin 1 is not expressed in the brain^57^. While our observed abnormalities here in the Rab27 DKO mouse with the additional αsyn deletion are likely more severe than in Rab27 KO or αsyn KO models, a direct comparison of the combined loss of these genes to single gene loss would need to be done to fully understand this potential interplay. This mouse could be useful for examining the role of these three proteins in synaptic function.

In conclusion, we observed that the previously described Rab27 DKO mouse has a spontaneous deletion in chromosome 6 involving additional genes, including *Snca* and *Mmrn1*. This DKO mouse has been used to attribute Rab27a and Rab27b to many trafficking functions, yet it is possible that some of these functional links to Rab27s could be related to deletions of *Snca* and/or *Mmrn1*. Mouse models are essential to the furthering of knowledge due to disease states, yet uncharacterized genetic deletions or mutations in mice may unintentionally led to erroneous conclusions. Our findings here reinforce the importance of validating scientific findings by multiple models and approaches. Backcrossing of the Rab27 DKO mouse line to other C57BL/6J substrains is recommended to restore *Snca* and *Mmrn1* expression before using these mice for studies to examine the role of Rab27s.

## Methods

### Mice

Wildtype C57BL/6 mice were obtained from Jackson Laboratory. Rab27 DKO mice used in this study were obtained through a material transfer agreement with Miguel Seabra at Imperial College of London and Brian Rudd at Cornell University. Mice were maintained and used in accordance with the guidelines of the National Institute of Health (NIH) and University of Alabama at Birmingham (UAB) Institutional Animal Care and Use Committee (IACUC). The animal work performed in this study was approved by UAB’s IACUC. We complied with ARRIVE guidelines. Both male and female mice were used for the studies.

Rab27 DKO mice used in this study were obtained by breeding non-littermate Rab27 DKO mice to each other. Since this DKO mouse was generated in the C57BL/6J strain, we used C57BL/6J mice obtained from Jackson Laboratories that were bred to each other in our animal colony to demonstrate the loss of αsyn expression. Every few generations, new mice from Jackson Laboratories were obtained to breed into our C57BL/6J colony.

### Western blot

Cortex and hippocampi from 4 week-old wildtype C57BL/6J mice and Rab27 DKO mice were dissected, homogenized with a motorized tissue grinder, and sonicated in lysis buffer (175 mM NaCl, 50 mM Tris–HCl, pH 7.4, 5 mM EDTA, protease inhibitor cocktail [ThermoFisher Scientific]). After 1% Triton X-100 was added, and after a 30 min incubation on ice, samples were centrifuged at 15,000*g* × 60 min at 4 °C. Supernatant was the Triton X-100 soluble fraction. Pellets were resuspended in lysis buffer with 2% SDS, sonicated for 10 s, and then spun at 15,000*g* for 10 min. Supernatant was the Triton X-100 insoluble fraction. Western blot analysis was performed as described previously^45^. Briefly, equal total protein was loaded per sample, resolved on a 12% SDS–polyacrylamide gel, and then transferred to nitrocellulose membranes. Membranes were blocked in 5% nonfat dry milk solution followed by incubation in primary antibody (αsyn antibodies: Becton Dickinson #610787 and Cell Signaling Technology #2642S; β-actin antibody ThermoFisher Scientific MA1-140) at 4 °C overnight. Membranes were then incubated in HRP-conjugated goat anti-mouse secondary antibody (Jackson ImmunoResearch) and developed with the enhanced chemiluminescence method. Images were scanned using the Bio-Rad Chemidoc Imaging System.

### RNA extraction and reverse transcriptase PCR

RNA was extracted from the cortex and hippocampi of wildtype C57BL/6J mice and Rab27 DKO mice using the Qiagen RNAeasy kit, according to the manufacturer’s protocol, and reverse transcribed into first-strand cDNA using the SuperScript III Reverse Transcriptase Kit (Invitrogen). Primers were designed using Primer3 (http://frodo.wi.mit.edu), and primer sequences are described in Table 1. PCR was performed using a BioRad MyCycler set to the following protocol: 1 cycle of denaturation at 94 °C for 3 min; 35 cycles of denaturation at 94 °C for 30 s, annealing at 55 °C for 30 s, and polymerization at 72 °C for 30 s; and 1 cycle of extension for 1 min.

**Table 1:** Primers used for RT-PCR and genomic PCR.

Gene	Sequence
*Actin*	Forward: TCCTGACCGAGCGTGGCTAC Reverse: CGGAACCGCTCGTTGCCAAT
*Atoh1*	Forward: CTGCAGGCGAGAGACCTTC Reverse: TCAGCTTGCACAGCT GTTC
*Atoh2*	Forward: TACTGCAGTGCATATGAATC Reverse: TCGTAAGGGAAGTGGCTGTC
*αsyn *(*for RT-PCR*)	Forward: AGGAGTGGTTCATGGAGTGA Reverse: CACAGGCATGTCTTCCAGG
*αsyn *(*Exon 4*)	Forward: AGAAGACCAAAGAGCAAGTGACA Reverse: ATCTGGTCCTTCTTGACAAAGC
*αsyn *(*Exon 6*)	Forward: AAGACTATGAGCCTGAAGCCTAAG Reverse: AGTGTGAAGCCACAACAATATCC
*Beta-globin*	Forward: CCAATCTGCTCACACAGGATAGAGAGGGCAGG Reverse: CCTTGAGGCTGTCCAAGTGATTCAGGCCATCG
*Gm43894*	Forward: GGCCTGTCTTGCCTTCCA Reverse: TCCCTCAGTCCTGGACACTG
*Gm43896*	Forward: GGCCTGTCTTGCCTTCCATA Reverse: AGGGCCCAAGTCATACAAAGT
*Gm44410*	Forward: TCACTAAAGAAATGTGGGACGA Reverse: GGCTCCAATTTAGGGCATGC
*Gm5570*	Forward: CTCAGGGGTCCCAGATGGTA Reverse: CCTCCCGAGTCCCAACATTC
*Grid2*	Forward: TCTTCCTACAGGGAGGGTCG Reverse: GGGGAATCCTCCAGAGACCT
*Mmrn1*	Forward: CCCTGCCCTTCTAAGTCACG Reverse: GGATGAGAACCTGCCCAGTC

### Genomic DNA isolation

Tail tissue sample was incubated in tail digestion buffer (50 mM KCL, 10 mM Tris HCl, pH 9, 0.1% Triton X100) plus protease inhibitors with Proteinase K Plus enzyme at 55 °C overnight. Digested tail tissue was then incubated at 100 °C for 15 min to inactivate proteinase K.

### Genomic PCR

Extracted tail DNA was used examine whether the *Snca* locus and other genes around the *Snca* locus were present in genomic DNA from wildtype C57BL/6J mice and Rab27 DKO mice. To assess the *Snca* locus, we used previously developed site-directed primers for *Snca* exon 4 and exon 6^46^ (Table 1). PCR for exon 4 and exon 6 were performed using MyCycler set to the following protocol: 1 cycle of denaturation at 95 °C for 5 min; 30 cycles of denaturation at 94 °C for 30 s, annealing at 68 °C for 30 s with dT = − 0.5 °C each cycle, and polymerization at 72 °C for 45 s; and 10 additional cycles of denaturation at 94 °C for 30 s, annealing at constant 52 °C, and polymerization at 72 °C for 45 s, as previously described^46^.

Additional primers were designed for genes surrounding the *Snca* locus, including *Atoh2*, *Gm5570*, *Gm44410*, *Mmrn1*, *Gm43894*, *Gm43896*, *Grid 2*, *Gm44072*, and *Atoh 1* (Table 1). PCR for these genes and beta-globin were performed using MyCycler set to the following protocol: 1 cycle of denaturation at 94 °C for 3 min; 35 cycles of denaturation at 94 °C for 30 s, annealing at 55 °C for 30 s, and polymerization at 72 °C for 30 s; and 1 cycle of extension for 1 min.

### Whole genome sequencing

Extracted Tail DNAs were purified using Phenol Chloroform purification method and processed for whole genome sequencing using the Illumina Nextera XT kit following the manufacturer’s protocol for the library prep. Sequencing was done on the NextSeq 500 with 150 bp paired end sequencing reads as per standard protocols. Raw sequence FASTQ files were trimmed to remove any primer adapter contamination using Trim Galore! (https://www.bioinformatics.babraham.ac.uk/projects/trim_galore/) version 0.6.6 (parameters used: --paired --nextseq 20 –trim1). Trimmed sequence reads were then aligned to the UCSC mouse mm10 reference genome using BWA mem version 0.7.17-r1188 (parameters used: -M -R)^58^. Aligned reads were then sorted and duplicates removed.

### Transmission electron microscopy

C57BL/6J mice and Rab27 DKO mice were perfused using ½ strength Karnovsky buffer (2% paraformaldehyde, 2% glutaraldehyde and 2 mM CaCl_2_ made in 0.1 M sodium cacodylate buffer). After dissection, brains were post fixed in the same solution for 6 h and then placed in 0.1 M sodium cacodylate buffer. 1 mm^3^ pieces of motor cortex were sectioned and fixed with osmic acid solution (1% osmic acid in 0.1 M Na-cacodylate buffer) for 3 h. The samples were then dehydrated using increased ethanol concentrations ranging from 50 to 100% for 3 × 5 min for each ethanol concentration. The samples were then incubated with Propylene oxide for 10 min and then again for 15 min, plastic/propylene oxide mixture (1:1 V/V) for 1 h, plastic/propylene oxide (2:1 V/V) overnight. After drying, samples were embedded in plastic overnight at 60 °C before sectioning. The plastic mixture was composed of 50% dodecenyl succinic anhydrite, 33% Araldite 6005, 11% Embed 812 Resin, 1.7% dibutyl phthalate with 50 drops of benzyldimethyl amine. Thin sections were taken with a diamond knife using a Leica Ultracut-S microtome and stained with uranyl acetate and lead citrate. Imaging was done using an FEI Tecnai-Spirit electron microscope with an AMT digital camera.

Synapses were analyzed at 11,000 × magnification by a researcher blinded to the experimental conditions. Briefly, synapses were identified by the presence of a clear active zone and postsynaptic density. For each synapse, the number of small, clear (< 60 nm) synaptic vesicles and the presynaptic bouton area were measured in Fiji (ImageJ), from which the vesicle density was calculated. All statistical analyses and graphing were performed in Prism 9.2.0 (GraphPad Software, Inc., La Jolla, CA).

## Supplementary Information

Supplementary Figures.

## Data Availability

Whole genome sequencing data is available through the Sequence Read Archive database at the National Library of Medicine, accession #PRJNA818694.
