# Supplementary material for: Deletion in chromosome 6 spanning alpha-synuclein and multimerin1 loci in the Rab27a/b double knockout mouse
Source: Sci Rep. 2022 Jun 14;12:9837. doi: 10.1038/s41598-022-13557-8 (PMC9197848; doi:10.1038/s41598-022-13557-8)
Supplement: Supplementary file 1 — Supplementary Figures. [file 41598_2022_13557_MOESM1_ESM.pdf]

Supplemental Materials

Supplemental Figure 1. Rab27 DKO mouse brains lack αsyn protein expression.

a. Full-length, unprocessed Western blots of total cortical or hippocampal homogenates from Figure 1b.

b. Full-length, unprocessed Western blots of Triton X-100 soluble and insoluble cortical lysates from Figure 1c.

Supplemental Figure 2. Rab27 DKO mouse brains lack αsyn mRNA expression.

a. Full-length, unprocessed PCR gel of RT-PCR from cortical samples from Figure 1d.

b. Full-length, unprocessed PCR gel of RT-PCR from hippocampal samples from Figure 1d.

Supplemental Figure 3. Rab27 DKO mice lack *Snca* and *Mmrn1* loci on chromosome 6.

a. Full-length, unprocessed PCR gel for *Snca* from genomic DNA from Figure 2a.

b. Full-length, unprocessed PCR gel for loci surrounding *Snca* from genomic DNA from Figure 2b.

SF1

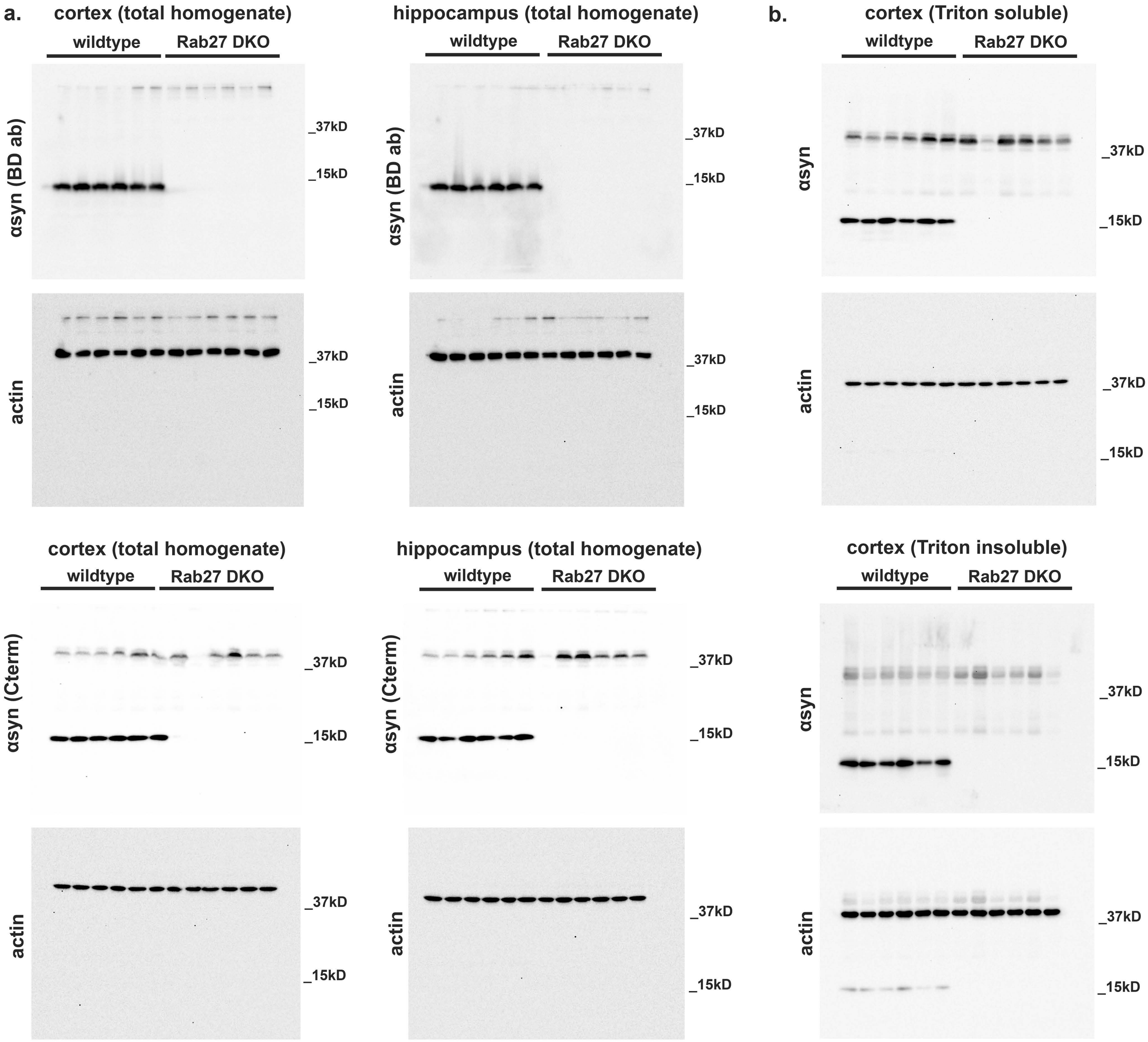
a.
cortex (total homogenate)
wildtype
Rab27 DKO
αsyn (BD ab)
_37kD
_15kD
actin
_37kD
_15kD
hippocampus (total homogenate)
wildtype
Rab27 DKO
αsyn (BD ab)
_37kD
_15kD
actin
_37kD
_15kD
cortex (total homogenate)
wildtype
Rab27 DKO
αsyn (Cterm)
_37kD
_15kD
actin
_37kD
_15kD
hippocampus (total homogenate)
wildtype
Rab27 DKO
αsyn (Cterm)
_37kD
_15kD
actin
_37kD
_15kD
b.
cortex (Triton soluble)
wildtype
Rab27 DKO
αsyn
_37kD
_15kD
actin
_37kD
_15kD
cortex (Triton insoluble)
wildtype
Rab27 DKO
αsyn
_37kD
_15kD
actin
_37kD
_15kD

SF2

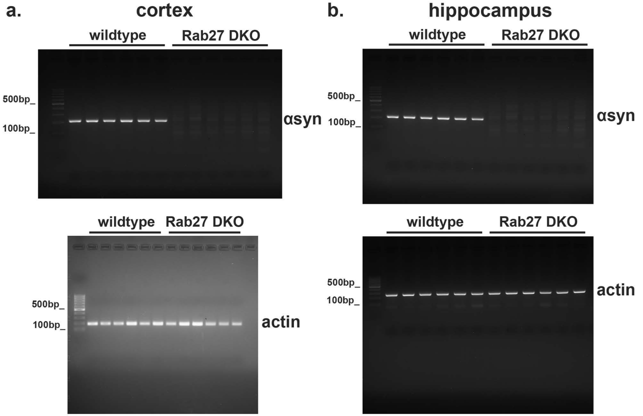
a.
cortex
wildtype
Rab27 DKO
500bp_
100bp_
αsyn
wildtype
Rab27 DKO
500bp_
100bp_
actin
b.
hippocampus
wildtype
Rab27 DKO
500bp_
100bp_
αsyn
wildtype
Rab27 DKO
500bp_
100bp_
actin

SF3

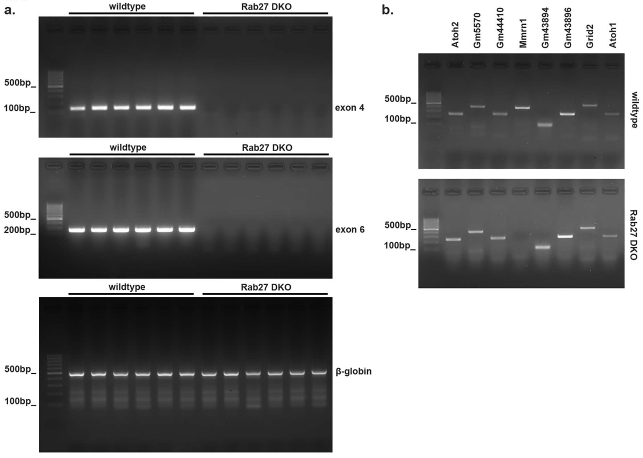
a.
wildtype
Rab27 DKO
500bp_
100bp_
exon 4
wildtype
Rab27 DKO
500bp_
200bp_
exon 6
wildtype
Rab27 DKO
500bp_
100bp_
β-globin
b.
Atoh2
Gm5570
Gm44410
Mmrn1
Gm43894
Gm43896
Grid2
Atoh1
500bp_
100bp_
wildtype
500bp_
100bp_
Rab27 DKO
